# pH-Responsive Nanoparticles for Cancer Immunotherapy: A Brief Review

**DOI:** 10.3390/nano10081613

**Published:** 2020-08-17

**Authors:** Yunfeng Yan, Hangwei Ding

**Affiliations:** College of Biotechnology and Bioengineering, Zhejiang University of Technology, Hangzhou 310032, China; dhwpao@163.com

**Keywords:** nanoparticle, cancer, immunotherapy, pH-responsive, drug delivery

## Abstract

Immunotherapy has recently become a promising strategy for the treatment of a wide range of cancers. However, the broad implementation of cancer immunotherapy suffers from inadequate efficacy and toxic side effects. Integrating pH-responsive nanoparticles into immunotherapy is a powerful approach to tackle these challenges because they are able to target the tumor tissues and organelles of antigen-presenting cells (APCs) which have a characteristic acidic microenvironment. The spatiotemporal control of immunotherapeutic drugs using pH-responsive nanoparticles endows cancer immunotherapy with enhanced antitumor immunity and reduced off-tumor immunity. In this review, we first discuss the cancer-immunity circle and how nanoparticles can modulate the key steps in this circle. Then, we highlight the recent advances in cancer immunotherapy with pH-responsive nanoparticles and discuss the perspective for this emerging area.

## 1. Introduction

Immunotherapy has revolutionized the cancer treatment by activating the innate and adaptive immune system against tumor cells with immune checkpoint inhibitors (ICIs), agonists, antigens, or engineered T cells. In contrast to the conventional cancer treatment modalities, e.g., chemotherapy, radiotherapy, and surgery, which directly kill cancer cells or resect tumor tissues, immunotherapy aims to restore the antitumor activity of the immune system to attack abnormal cells through natural mechanisms, allowing better potency and fewer off-target effects in the treatment of advanced malignancies [1,2,3,4]. Several notable clinical successes in cancer immunotherapy have been made over the past decade, including the FDA approval of the chimeric antigen receptor (CAR) T cell therapy and therapies with monoclonal antibodies (mAbs) targeting cytotoxic T lymphocyte antigen 4 (CTLA4), programmed cell death 1 (PD-1), or its ligand (PD-L1) as the immune checkpoint inhibitors. Due to their contributions in the discovery of cancer therapy through the immune checkpoint blockade, the Nobel Prize in Physiology or Medicine 2018 was awarded to James P. Allison and Tasuku Honjo. Now, there is a large number of active clinical trials worldwide and immunotherapy has become a new pillar of cancer treatment owing to these tremendous achievements [5]. 

### 1.1. Modulation of Anticancer Immunity

**Figure 1:**
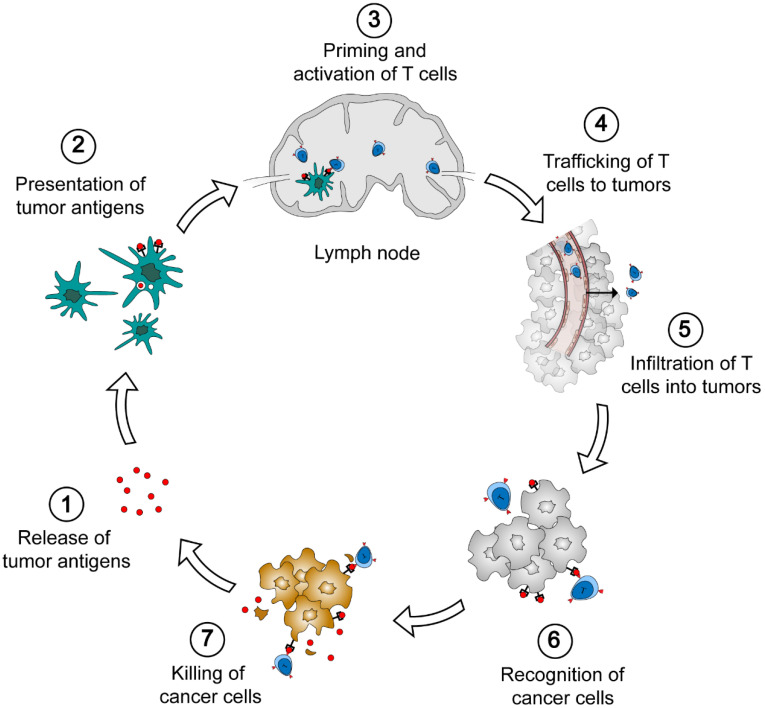
The cancer-immunity cycle. Seven steps are involved in the generation of antitumor immunity, i.e., the release of tumor antigens from cancer cells, the presentation of tumor antigen to antigen-presenting cells (APCs), the priming and activation of T cells in lymph nodes, the trafficking and infiltration of T cells to tumors, the recognition and the killing of cancer cells.

The generation of endogenous immune response against tumors involves several distinct steps (Figure 1) [1,6,7]. First, tumor-associated antigens (TAAs) are released from cancer cells. The immunogenic signal could be proinflammatory cytokines or factors which are produced in oncogenesis (step 1). Then, TAAs are captured by antigen presenting cells (APCs) (e.g., dendritic cells) and presented on major histocompatibility complex (MHC) class I and class II molecules (step 2). Tumor antigen-loaded dendritic cells migrate to lymph nodes, resulting in the priming and activation of T cells (step 3). This is a crucial step to generate T cell response against cancer-specific antigens. In the presence of immunogenic stimulators, the antigen presentation elicits effector T cells which are killers to cancer cells. Without such stimulation, dendritic cells will induce T cell deletion and the production of regulatory T cells which relate to the immunosuppression. The activated T cells subsequently leave the lymph node, traffic to tumors through the bloodstream, and infiltrate into tumor parenchyma (steps 4 and 5). Finally, effector T cells recognize cancer cells by the specific binding of T cell receptor (TCR) to the antigen on cancer cell surface (step 6), and kill target cancer cells (step 7). The death of cancer cells further promotes the release of TAAs and elicits the subsequent immune response.

Durable cancer immunotherapy requires the complete cancer-immunity cycle. However, a few factors may waken or suspend the generation and performance of antitumor immunity in cancer patients. The approaches to overcome these obstacles derive the major classes of cancer immunotherapy. The cancer antigens may not be sufficiently released from solid tumors and captured by dendritic cells for the further processing. In this respect, treatments with conventional chemotherapeutics or radiation induce the apoptosis of cancer cells that promotes the release of tumor antigens from dead cells [5]. The immunization could be also initiated with the delivery of exogenous vaccines including conventional protein or peptide antigens, nucleic acids, and dendritic cells. The tumor-associated proteins, peptides, and nucleic acids could be directly administered to cancer patients, then, processed by dendritic cells and cross-presented to T cells in vivo. Alternatively, dendritic cells are engineered with specific antigens ex vivo and injected into patients for personalized immunization [8]. Stimulatory molecules are requisite for dendritic cell maturation, antigen presentation, and T cell activation. For example, agonists of Toll-like receptor (TLR), e.g., cytosine-phosphate-guanine oligonucleotide (CpG-ODN) for TLR9 and Imiquimod for TLR7, and stimulator of interferon genes (STING) are able to promote the maturation of APCs [9,10]. Incubation with interferon-α (IFN-α) and granulocyte-macrophage colony-stimulating factor (GM-CSF) facilitates dendritic cell development and the expression of leukocyte antigen (HLA), B7 co-stimulatory molecules, MHC proteins, and CD40, which benefits the TAAs presentation and immunization [11]. Interleukin-2 (IL-2) is able to stimulate the expanding and activation of T cells in lymph nodes [12]. These cytokines and agonists are legitimately utilized to improve the immune activity of T cells for cancer immunotherapy. To circumvent the elaborate procedures of T cell priming and activation in vivo, T cells are collected from tumors or peripheral blood, selected, genetically engineered, and proliferated in vitro, followed by the reinfusion into the tumor-bearing patient [13]. This strategy of adoptive T cell therapy represents a major advancement of cancer immunotherapy in the past decade. In particular, T cells modified with chimeric antigen receptor (CARs) show exceptional immune activity which have been approved for clinical use to treat B cell acute lymphoblastic leukemia and B cell non-Hodgkin lymphoma [14,15]. 

Abnormal angiogenesis and proliferation of cancer cells and cancer-associated fibroblasts (CAFs) contribute to the formation of solid tumors with high interstitial fluid pressure (IFP) that hinders the infiltration of all therapeutics into tumor parenchyma from blood vessels [16]. In addition to physical barriers, reduced blood flow and substance exchange further induce the hypoxia and acidity in tumor, resulting in the immunosuppressed tumor microenvironment (TME) that is a major cause of the resistance to the current cancer immunotherapy. The therapeutics for the normalization of TME, e.g., antiangiogenic and CAF-reprogramming agents, have been broadly utilized to improve the tumor perfusion and immunity for cancer immunotherapy [1]. The activity of effector T cells could be suppressed by immunosuppressive macrophages in the TME, including regulatory T cells, M2-like tumor-associated macrophages (TAMs), and myeloid-derived suppressor cells because they are able to secrete a number of immunosuppressive factors (e.g., NO, reactive oxygen species, arginase, iterleukin-10, indoleamine 2,3-dixoygenase, and transforming growth factor-β) and to down-regulate the cytotoxicity of effector T cells [5]. Reprogramming or eliminating immunosuppressive cells has proven to be a complementary approach to augment the antitumor immunity of T cells in solid tumors. Cancer cells usually express immune checkpoint proteins on the surface, leading to the immune resistance when these proteins bind to the specific ligands on T cells. Inhibition of the immune checkpoints with anti-CTLA4, anti-PD-1, or anti-PD-L1 antibodies represents the most notable approach in the current cancer immunotherapy [2]. There are now at least six FDA-approved immune checkpoint inhibitors for the treatment of a wide range of cancers [3]. 

### 1.2. Ongoing Challenges in Cancer Immunotherapy

Despite the substantial progress in recent years, the broad implementation of cancer immunotherapy remains challenging. The response rate and magnitude of cancer patients to immunotherapies remains moderate. Only <13% of cancer patients effectively respond to the current immune checkpoint inhibitors because the expression level of checkpoint proteins varies with cancer types and patients [17]. CAR-T cell therapy shows high potency for the treatment of hematologic malignancies. However, its clinical application to solid tumors is still unfulfilled due to the compact and immunosuppressive microenvironment of solid tumors [18]. In addition to the unsatisfactory efficacy, the safety issues further limited the broader clinical use of immunotherapeutics. CAR-T therapy requires successive infusion of CAR-T cells that may cause severe side effects including cytokine-release syndrome (CRS) and CAR-T cell-related encephalopathy syndrome (CRES) [18]. The advance of immune checkpoint inhibitors (ICI) therapy is also associated with some immune-related adverse events (irAEs) including colitis, pneumonitis, hepatitis, myocarditis, and neurotoxic effects [19]. Some immune modulators are toxic and the repeated administration leads to accumulative toxicity for the patients. In addition, the stimulating and activating circulating lymphocytes out of tumors may lead to the attack on normal tissues, causing the off-target side effects [2,19]. The ideal cancer immunotherapy should be capable of precise modulation on the strength and the site of immune response to optimize the clinical outcomes. 

## 2. Cancer Immunotherapy with Nanoparticles

Nanoparticles, particles with a typical size of 1–100 nm in diameter, have been widely utilized in cancer treatments [2,16,20]. Nanoparticle-based delivery offers potent approaches for the spatiotemporal control of immunotherapeutic agents to reduce the adverse effects and maximize the therapeutic index of cancer immunotherapy [1,2,3,4,5]. First, the formulation of immunotherapeutics in nanoparticles can improve the pharmacological properties of drugs, including the solubility and stability. Of particular importance, some biologic drugs, e.g., nucleic acids and antibodies, require protection from degradation and macrophage clearance in blood after systemic administration. Second, nanoparticle platforms enable versatile modification or functionalization to modulate the pharmacokinetic profile of drugs and regulate the interaction between drugs and cells or organs. The level of antibodies or small drugs can be tuned by controlled release to extend the efficacy and avoid the systemic toxicity due to the instantaneous high concentration after systemic administration. In addition, the structure of nanoparticles can be readily designed for the active targeting and the smart response to external stimuli (e.g., light, electronic, and magnetic fields) or the biochemical changes from normal tissues to tumors (e.g., pH, redox potential, and enzymes), resulting in the enhanced tumor accumulation and reduced off-target side effects. For the highly hydrophilic and negatively charged nucleic acid drugs, nanoparticle carriers play significant roles in their cellular uptake, endosomal escape, and release in target cells that are the critical steps for the implementation of nucleic acid-based immunotherapy. Finally, nanoparticle technologies allow feasible combinations of immunotherapy with conventional chemotherapy, radiotherapy, as well as photothermal and photodynamic therapy for the normalization of immunosuppressive TME and improved immunotherapy efficacy. 

## 3. pH-Responsive Nanoparticles for Cancer Immunotherapy

pH-responsive nanoparticles have received intensive attention in cancer immunotherapy because of the distinct acidic features of a tumor microenvironment compared with normal tissues. Deregulated glycolysis in cancer cells results in the high level of lactic acid and consequent acidic pH (6.5–6.9) in tumor tissues [21]. pH-sensitive nanoparticles afford cancer immunotherapy improved pharmacology and enhanced accumulation of immunotherapeutics in tumor tissues. In addition, the intracellular trafficking of drug nanoparticles usually undergoes early endosomes, late endosomes, and fusion with lysosomes with a decreased pH from 6.5 to 4.5 [22,23]. The formulation of biologic drugs with pH-sensitive carriers is able to response to the subtle pH change, facilitating the endosomal escape and avoiding the degradation of nucleic acids or proteins in lysosomes. It is worth noting that pH-responsive nanoparticles have been extensively used in other cancer therapies (e.g., chemotherapy) besides immunotherapy. For these topics, readers may refer to the published reviewer articles [24,25,26]. This review will focus on the recent advances in cancer immunotherapy using pH-responsive nanoparticles and offer perspectives on this burgeoning field.

**Figure 2:**
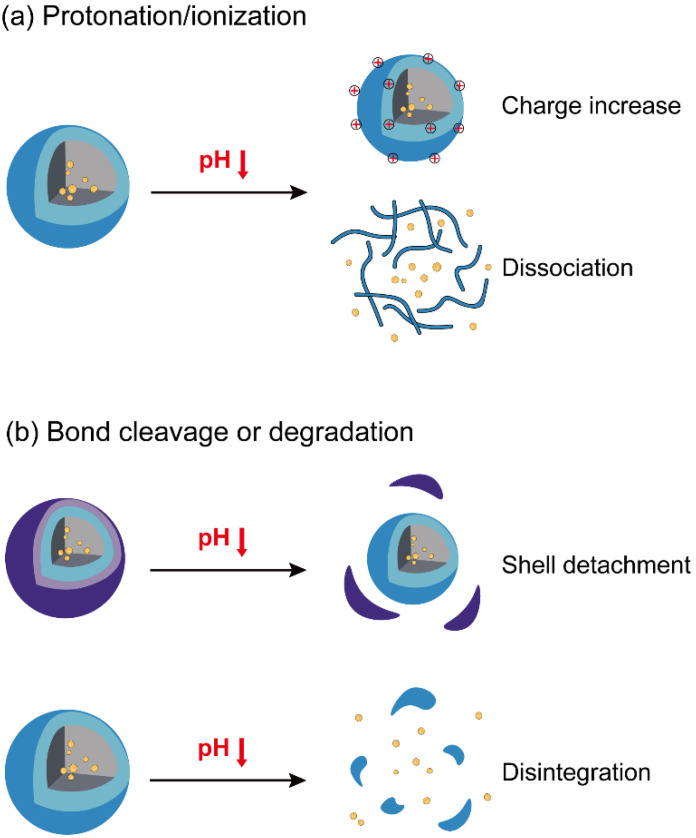
The typical approaches to the pH-response of nanoparticles for cancer immunotherapy. (**a**) Protonation/ionization and (**b**) cleavage of acid-labile shells or degradation of nanoparticles at acid pH enables the significant change of surface properties or the disintegration of nanoparticles.

Nanoparticles’ response to pH change in two typical ways: the protonation/ionization of functional groups and the degradation or the cleavage of acid-labile bonds (Figure 2). pH-dependent protonation/ionization is frequently utilized in the design of pH-triggered delivery systems. In this strategy, various ionizable groups (e.g., amines and carboxyl acids) are incorporated into the delivery carriers, endowing drug-loaded nanoparticles with sensitivity to acid environments. pH variation alters the protonation of amines or the ionization of carboxyl groups that result in the change of the surface charge, the stability, and the interaction with cells or tissues. The pH-dependent protonation of carriers enables the “proton sponge” effect in the intracellular trafficking of nanoparticles which is crucial for the endosomal escape of biological immunotherapeutics (e.g., nucleic acids and proteins) [23]. The protonation of amines increases with the acid degree in endosomes, leading to the extensive influx of water and counterions into the late endosomes, which further results in the rapture of the endosomal membrane and the release of cargoes into the cytoplasm, therefore, avoiding the degradation of the biological drugs in lysosomes. In contrast to the physical changes in pH-responsive protonation/ionization, the increase of acidity may lead to the break of covalent bonds including amide, ester, imine, oxime, acetal, and ketal bonds or the disintegration of inorganic components, altering the physicochemical properties of drug-loaded nanoparticles. For example, acid cleavable PEG segments are incorporated into nanocarriers to shield nanoparticles from protein adsorption and aggregation under physiological conditions. When nanoparticles are circulated to the acidic tumors, the acid-labile linkers break and shielding PEGs are detached from nanoparticles, shifting the surface from a hydrophilic to a hydrophobic one or from a neutral to a positive one that improves the accumulation and retention of nanoparticles in tumor tissues [27,28]. In contrast to changes on the surface, the hydrolysis inside the nanoparticles triggers the degradation and disassembly of nanoparticles under acidic conditions, leading to the rapid release of immune cargoes and facilitating the antigen presentation in the target tissue [29,30]. Several pH-responsive inorganic components have been utilized to construct nanocarriers for cancer immunotherapy because they are highly sensitive to pH change from a physiological to a tumor environment [30,31].

### 3.1. Nanoparticles with pH-Responsive Protonation/Ionization for Cancer Immunotherapy

**Figure 3:**
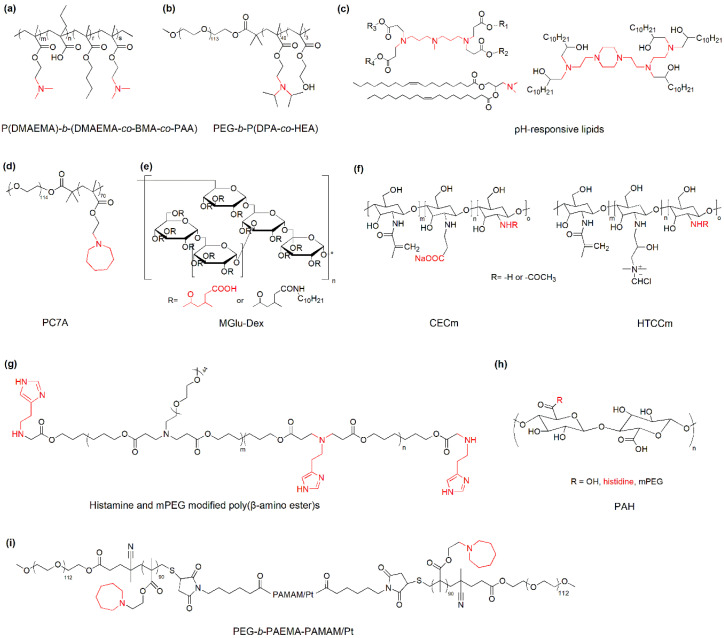
Representative materials with pH-dependent protonation/ionization for cancer immunotherapy. (**a**) P(DMAEMA)-*b*-(DMAEMA-*co*-BMA-*co*-PAA): poly(dimethylaminoethyl methacrylate)-*b*-(dimethylaminoethyl methacrylate-*co*-butyl methacrylate-*co*-propylacrylic acid); (**b**) PEG-*b*-P(DPA-*co*-HEA): poly(ethylene glycol)-*b*-poly(diisopropanol amino ethyl methacrylate-*co*-hydroxyethyl methacrylate); (**c**) pH-responsive lipids; (**d**) PC7A: poly(ethylene glycol)-*b*-poly(2-(hexamethyleneimino)ethyl methacrylate); (**e**) MGlu-Dex: dextran derivatives having 3-methylglutarylated residues; (**f**) CECm: amphoteric methacrylamide *N*-carboxyethyl chitosan; HTCCm: methacrylamide *N*-(2-hydroxy)propyl-3-trmethylammonium chitosan chloride; (**g**) histamine and mPEG modified poly(*β*-amino ester)s; (**h**) PAH: PEG-histidine modified alginate; and (**i**) PEG-*b*-PAEMA-PAMAM/Pt: platinum (Pt)-prodrug conjugated and poly(ethylene glycol)-*b*-poly(2-azepane ethyl methacrylate)-modified polyamidoamine. The key pH-sensitive groups are indicated in red.

pH-responsive polycations (e.g., poly(2-diethylamino ethyl methacrylate), PDEAEMA) take positive charges upon the protonation of amine groups and enable the proton sponge effect after internalization into cells (Figure 3). Therefore, they could be used to encapsulate negatively charged immunotherapeutic nucleic acids, improve their cellular uptake, and protect intrinsically unstable nucleic acids from degradation in acidic lysosomes in dendritic cells [32]. For example, poly(dimethylaminoethyl methacrylate)-*b*-(dimethylaminoethyl methacrylate-*co*-butyl methacrylate-*co*-propylacrylic acid) (P(DMAEMA)-*b*-(DMAEMA-*co*-BMA-*co*-PAA)) (Figure 3a) was utilized for the delivery of a RNA agonist of the retinoic acid gene (3pRNA) to dendritic cells. The pH-responsive polymer-nucleic acid nanoparticles reduce nuclease degradation and improve cellular uptake and endosomal escape of 3pRNA, enhancing the immunostimulatory activity and the therapeutic efficacy of anti-PD-1 immune checkpoint blockade in a CT26 colon cancer model [33]. Similar pH-responsive polymers, poly(ethylene glycol)-*b*-poly(diisopropanol amino ethyl methacrylate-*co*-hydroxyethyl methacrylate) (PEG-*b*-P(DPA-*co*-HEA)) (Figure 3b) and 1,2-epoxytetradecane alkylated oligoethylenimine (OEI-C14) were utilized to deliver a photosensitizer (PS) and small interfering RNA against PD-L1 (siPD-L1) for combination of photodynamic therapy and RNA interference (RNAi)-based PD-L1 blockade [34]. At physiological pH, the carriers and payloads form stable nanoparticles wherein the fluorescence of photosensitizers is quenched due to the fluorescence resonance energy transfer, implying reduced dark toxicity in the blood circulation upon laser irradiation. After entering the weakly acidic endocytic vesicles in tumor cells (pH 5.0–6.0), the protonation of the tertiary amines of PDPA increases, resulting in the dissociation of nanoparticles and the release of photosensitizers into tumor cells that mediates photodynamic immunotherapy with laser irradiation. Furthermore, the photodynamic treatment induces tumor-specific reactive oxygen species (ROS) and promotes the release of antigens, stimulating the adaptive anti-tumor immunity. The combination of photodynamic immunotherapy and PD-L1 knockdown on the acid-activatable nanoplatform significantly inhibits tumor growth and metastasis in a B16-F10 melanoma xenograft tumor model.

Messenger RNA (mRNA) has great potential in cancer immunotherapy [35]. A successful delivery of TAAs-encoded mRNA into DCs enhances the antigen presentation and the tumor specific immune response. In comparison with a short double-stranded RNA, a single-stranded mRNA is much longer, more flexible, and less stable. Further application of mRNA in cancer immunotherapy requires robust delivery carriers [36]. pH-responsive lipid nanoparticles (Figure 3c) are proven carriers for the cellular uptake and the endosomal escape of mRNA both in vitro and in vivo [17]. There is not a universal carrier for the delivery of any RNAs in different tumor models. In general, a proper content of pH-responsive amines and a delicate balance between hydrophilicity and hydrophobicity is needed to tackle the complicate challenge in RNA delivery. Of particular importance, amines with a p*K*_a_ of 6.0–6.5 are crucial for the binding, cellular uptake, and the release of RNAs. A recent research shows that the alteration of the component of lipid nanoparticles changes the global apparent p*K*_a_ and the protein corona of nanoparticles that enables the selective delivery of RNAs and proteins to target cells and tissues [37].

Ultra-pH-sensitive nanoparticles consisting of copolymers containing varying tertiary amines have been developed for the delivery of protein antigens to APCs in draining lymph nodes. Due to the robust response to the subtle pH change in organelles, the leading nanoparticle PC7A (Figure 3d) enables excellent cytosolic delivery and efficient surface presentation of tumor antigens, generating a strong cytotoxic T cell response with low systemic cytokine expression [38]. 

Carboxyl groups have been incorporated into pH-responsive nanoparticles for cancer immunotherapy because their pH-dependent ionization enables the change of hydrophilicity/hydrophobicity of carriers and the modulation of drug release from nanoparticles at varying pHs. For example, dextran was functionalized with carboxyl pendants and C12 alkyl side chains for the fabrication of pH-responsive liposomes for the delivery of a model antigen, ovalbumin (OVA) (Figure 3e). The modified liposomes are stable at neutral pH but destabilized at weakly acidic pH because the solubility of carboxy-bearing dextran decreases with pH, enhancing the release of OVA in the cytosol of dendritic cells. The pH-sensitive OVA-loaded liposomes demonstrate significant suppression of tumors upon subcutaneous injection to E.G7-OVA tumor-bearing mice [39].

In addition to improving the delivery efficacy at a cellular level, pH-responsive nanoparticles benefit cancer immunotherapy by enhancing the accumulation of immunotherapeutic drugs or targeting TAMs in tumor tissues through the charge change in acidic conditions. Paclitaxel (PTX) and interleukin-2 were encapsulated in nanogels composed of hydroxypropyl-*β*-cyclodextrin acrylate, red blood cell membrane, and two opposite charged chitosan to remodel the immunosuppressive tumor microenvironment (Figure 3f). With the pH decrease in the tumor environment, the ionization of –COOH decreases while the protonation of –NH_2_ increases, reversing the main driving force in nanogels from electrostatic attraction to repulsion, which further leads to the disintegration of the nanogel and the release of drugs in tumor tissues. The combinational chemotherapy and immunotherapy with the tumor microenvironment responsive nanogel significantly enhance the infiltration of immune effector cells and reduce the immunosuppressive factors in a murine melanoma model [40].

TAM is one of the key targets for the cancer immunotherapy besides tumor cells. Reversing TAMs from a tumor supportive phenotype to a tumoricidal phenotype is an effective way to remodel the immunosuppressive TME and enhance the antitumor immunity of immunotherapy. Histamine and mPEG modified poly(*β*-amino ester)s (Figure 3g) were prepared for the delivery of IL-12 to re-educate TAMs in TME. The drug-polymer nanoparticles swell under weak acidic conditions (e.g., pH 6.5), resulting in effective accumulation and prolonged release of IL-12 in TME that reverses the tumor-infiltrated macrophage phenotype from M2 to M1. This nanoparticle platform shows great potential in local re-education of TAMs in solid tumors with low systemic side effects in cancer immunotherapy [41]. In another report, pH-sensitive PEG-histidine modified alginate (PAH, p*K*_a_ ~ 6.9) (Figure 3h) was developed for the delivery of a combination of CpG oligodeoxynucleotide (ODN), anti-IL-10 ODN and anti-IL-10 receptor ODN, to alter the phenotype of TAMs and stimulate their antitumor immunity. Galactosylated cationic dextran was selected for the fabrication of a ODNs nanocomplex (GDO) for TAM targeting because of high level of galactose-type lectin on TAMs. GDO forms nanoparticles with PAH via electrostatic attraction at physiological pH. After entering the acidic TME, the charge of PAH changes from negative to positive, resulting the detachment of PAH from the GDO complex and the exposure of galactose for TAM targeting. The acidic tumor microenvironment-responsive and TAM-specific approach significantly reduces the systemic side effects of cancer immunotherapy by inhibiting the upregulation of serum proinflammatory cytokines [42].

Cancer therapy can be improved by targeting the delivery of chemotherapeutics and immune modulators to both TAMs and tumor cells. BLZ-945, a small molecule inhibitor of colony stimulating factor 1 receptor (CSF-1R) of TAMs, was encapsulated in ultra-pH-sensitive cluster nanoparticles (SCNs) which was constructed from the self-assembly of platinum (Pt)-prodrug conjugated and poly(ethylene glycol)-*b*-poly(2-azepane ethyl methacrylate)-modified polyamidoamine (PEG-*b*-PAEMA-PAMAM/Pt) (Figure 3i). At neutral pH, PAEMA is hydrophobic and maintains the stable nanoparticles for prolonged blood circulation and reduced systemic toxicity of payloads. PAEMA is rapidly protonated at tumor pH and becomes hydrophilic, leading to instantaneous disintegration of SCNs into small dendrimer nanoparticles (<10 nm) for deep tumor penetration and the release of BLZ-945 for TAM depletion. Comparing with BLZ-945 or Pt-loaded nanoparticles, the spatial targeting nanoparticles demonstrate better tumor growth suppression, metastasis inhibition, and mouse survival in multiple tumor models [43]. 

Silica nanoparticles with a pH-responsive surface have been used as scaffolds for the controlled release of drugs or enhanced accumulation in tumor tissues [28,31]. Mesoporous silica nanoparticles with pH- and GSH-responsive molecular gates were developed for doxorubicin (DOX) delivery for the treatment of metastatic tumors. The highly integrated nanoplatform demonstrates a robust response to the simultaneously acidic and reductive tumor microenvironment, enabling a precise release of drugs in tumor tissues. The smart nanoparticles not only show good chemotherapy efficacy but also stimulate the maturation of DCs and the release of antitumor cytokines [31]. Hollow silica nanoparticles were coated with PEG and 2-propionic-3-methylmaleic anhydride (CDM)-grafted PEI, enabling prolonged blood circulation and negative-to-positive charge conversion at acidic pH. The catalase and photosensitizer-loaded hybrid nanoparticles show enhanced retention in tumor tissue, leading to greatly relieved tumor hypoxia via decomposition of tumor endogenous H_2_O_2_ and improving anti-PD-L1 checkpoint blockade therapy [28].

### 3.2. Nanoparticles with pH-Responsive Bond Cleavage or Degradation for Cancer Immunotherapy

In addition to the physical change induced by protonation/ionization, the break of covalent bonds triggered by pH change can lead to the physicochemical property change on the surface of nanoparticles or the disintegration of the nanoparticle that mediates the targeting or the controlled release of payloads to tumors. 

**Figure 4:**
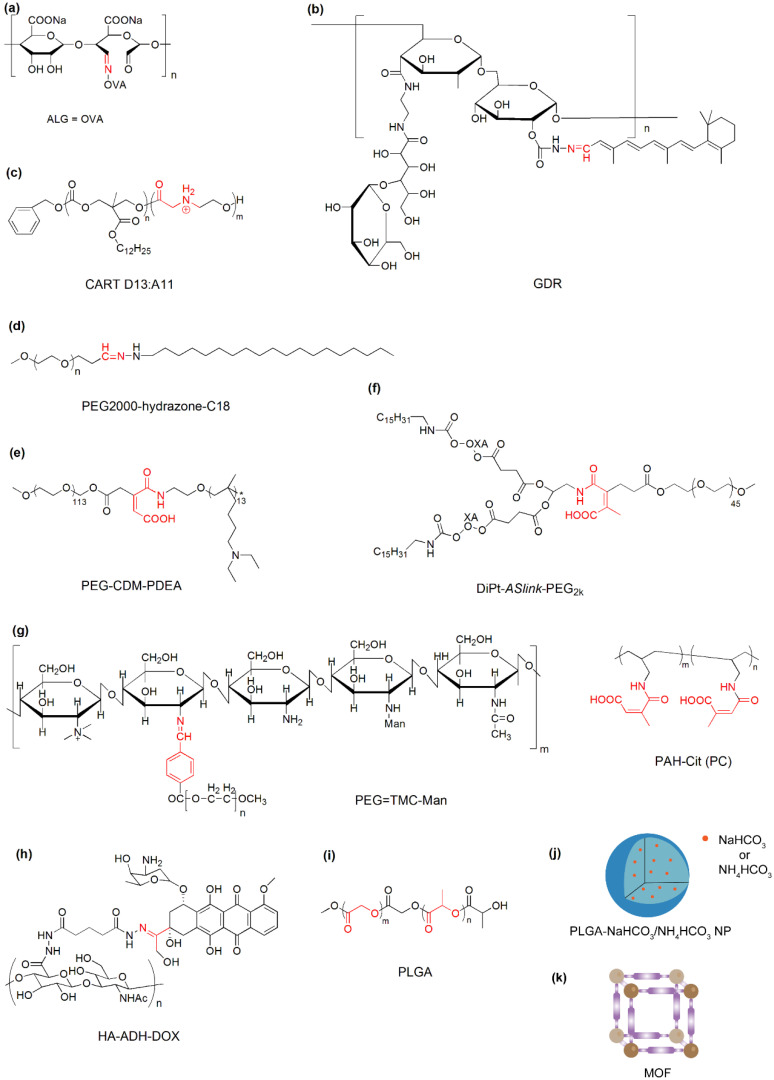
Representative materials with acid-labile bond cleavage or degradation for cancer immunotherapy. (**a**) ALG = OVA: ovalbumin-conjugated alginate; (**b**) GDR: galactosyl dextran-retinal conjugates; (**c**) CART D13:A11: charge-altering releasable transporter with 13 carbonate repeating units and 11 amino ester repeating units; (**d**) PEG2000-hydrazone-C18: conjugates of polyethylene glycol (molecular weight of 2000) and stearic hydrazide; (**e**) PEG-CDM-PDEA: conjugates of PEG and poly(2-(diethylamino) ethyl methacrylate with 2-propionic-3-methylmaleic anhydride linkers; (**f**) DiPt-*ASlink*-PEG_2k_: conjugates of PEG2000 and hexadecyl-oxaliplatin(IV) with 2-propionic-3-methylmaleic anhydride linkers; (**g**) PEG = TMC-Man: PEG and mannose doubly modified trimethyl chitosan; PAH-Cit (PC): citraconic anhydride-grafted poly (allylamine hydrochloride); (**h**) HA-ADH-DOX: conjugates of hyaluronic acid and doxorubicin with hydrazine linkers; (**i**) PLGA: poly(lactic-*co*-glycolic acid); (**j**) PLGA-NaHCO_3_/NH_4_HCO_3_ NP: NaHCO_3_ or NH_4_HCO_3_-encapsulated PLGA nanoparticle; and (**k**) MOF: Metal-organic framework. The acid-labile groups are indicated in red.

OVA was grafted to alginate (ALG) via pH-sensitive Schiff base bonds and formed nanovaccines with mannose modified ALG by CaCl_2_ crosslinking (Figure 4a). The nanovaccines are relatively stable at pH 7.4 and release OVA remarkably in the release in the endo/lysosomes (pH 4.5–5.5) due to the detachment of OVA from delivery vehicles after cleavage of a Schiff base linkage at acidic pH. Subcutaneous administration of the nanovaccines enables efficient trafficking of the OVA-bearing nanoparticles from the injection site to the draining lymph nodes, remarkably stimulating the major cytotoxic T lymphocytes (CTL) response and inhibiting E.G7 tumor growth in C57BL/6 mice [44]. Similarly, pH-responsive hydrazone bond has been utilized for the construction of alltrans retinal-loaded nanogels which show long-term stability at physiological pH but dissociate and release antigens in acidic lysosomes in DCs (Figure 4b) [29]. In a recent study, amphiphilic charge-altering releasable transporters (CARTs) (Figure 4c) were developed for mRNA delivery to multiple lymphocytes in which cationic poly(*α*-amino ester)s bind mRNA at acidic pH but release mRNA after a time-dependent rearrangement of poly(*α*-amino ester)s to neutral small molecules (diketopiperizine) at pH 7.4, resulting in enhanced lymphocyte transfection in primary T cells and in vivo in mice. In contrast to conventional polycations, CARTs provide a new mechanism for mRNA release and great potential to avoid polycation-associated tolerability issues [45].

Acid liable-PEG has been frequently used in pH-responsive nanoparticles to stabilize nanoparticles in the blood circulation while enhancing the nanoparticle accumulation in acidic tumor tissues. For example, nanoparticles composed of acid liable-PEG-hydrazone-C18 (PHC) (Figure 4d), poly(lactic-*co*-glycolic acid) (PLGA), and *O*-stearoyl mannose (M-C18) enable a decreased accumulation in the mononuclear phagocyte system (MPS) owing to the PEG shielding at normal pH, and thus reduce the off-target immune activation, while they can be effectively accumulated in TAM via mannose-receptor recognition after the hydrolysis of hydrazone bonds and the detachment of PEG in acidic TME [46]. 

The detachment of the PEG shell also enables the exposure of positively charged groups or negative-to-positive charge conversion on the nanoparticle surface [27,47]. The PEG block was conjugated with poly(2-(diethylamino) ethyl methacrylate (PDEA) using 2-propionic-3-methylmaleic anhydride (CDM) and formed mix micelles with PEI-PDEA for the delivery of siRNA against PD-L1 and a mitochondrion-targeting photosensitizer (Figure 4e). The detachment of the PEG corona at acidic pH endows nanoparticles with significant size reduction and surface charge increase in TME, facilitating the penetration of nanoparticles to tumors and improving the antitumor immune response in vivo. Using the same pH-sensitive linker, the PEG block was attached to a lipid with two tails (Figure 4f). The nanoparticle is negatively charged at neutral pH due to the partial ionization of carboxyl groups in the side chain. After entering acidic tumor tissue, the acid-labile linker was cleaved, resulting in the detachment of the PEG shell with negative carboxyl groups and the charge conversion from negative to positive. The smart nanoparticle shows enhanced tumor accumulation and deep penetration, consequently enabling efficient delivery of a combination of immunoregulators to suppress tumor growth and metastasis in mice [47].

Moreover, the cleavage of PEG induces simultaneous exposure of targeting ligands and positively charged groups that benefit cancer immunotherapy with enhanced accumulation and specific targeting to M2-TAMs or cancer cells in tumor tissues. For example, nanoparticles composed of PEG and mannose doubly modified trimethyl chitosan and citraconic anhydride-grafted poly (allylamine hydrochloride) (PC) have been developed for the delivery of siRNAs against the vascular endothelial growth factor (VEGF) and placental growth factor (PIGF) to breast cancer cells and M2-TAMs (Figure 4g). The PEG shell is able to mask mannose to reduce the uptake by resident macrophages in the reticuloendothelial system and improve the blood circulation time. When nanoparticles enter acidic tumor tissues, the benzamide bond between PEG and the mannose-modified trimethyl chitosan is cleaved, which results in the detachment of PEG and the exposure of mannose and cationic amines, enabling effective accumulation in tumor tissues and uptake by cancer cells and macrophages. In the more acidic late endosomes or lysosomes (pH 4.5–5.5), the side chains of PC are hydrolyzed, leading to the charge reversal from negative to positive and promoting the endosomal/lysosomal escape of siRNAs. The dual pH-responsive nanoparticle could be a robust platform to reverse TME from pro-oncogenic to anti-tumoral and suppress the tumor growth and metastasis [48].

A combination of an acid-cleavable bond and pH-dependent ionization has been utilized to construct a sequential pH-responsive delivery system for the co-delivery of a TLR7/8 agonist R848 and chemotherapeutic doxorubicin (DOX) (Figure 4h). Hyaluronic acid-DOX (HA-DOX) conjugates were prepared by coupling using acid-cleavable hydrazone bonds. R848 was bound with poly(L-histidine) (PHIS) and the PHIS/R848 nanocomplex were further coated with HA-DOX to form HA-DOX/PHIS/R848 nanoparticles. When pH decreases from neutral to acidic, the mixed nanoparticles undergo two distinguished changes. The ionization of PHIS at pH 6.5 leads to the hydrophobic-to-hydrophilic transition and the release of the encapsulated R848 in to TME. The cleavage of the hydrazone bond around pH 5.5 triggers the release of the covalently bound DOX to the cytosol of cancer cells, enabling the synergistic effects of immunotherapy and chemotherapy against breast cancer in 4T1 tumor-bearing mice [49].

In contrast to the cleavage of pH-responsive linkers, the break of acid-liable bonds in the polymer backbone induces the degradation of polymers and the disintegration of nanoparticles that facilitates the release of payloads into an acidic compartment of APCs or tumor tissues. For example, biodegradable PLGA-based nanoparticles have been used for the delivery of protein antigens (Figure 4i) (e.g., gp100, OVA) and JSI-124 (a small molecule inhibitor of activator of transcription-3, STAT3) to protect the immunoregulators and achieve the sustained release of drugs to DCs [50,51]. Biodegradable nanoparticles could be prepared by using acid degradable primary amine monomer and cross-linker in which anti-DEC-205 mABs were encapsulated for cytotoxic T lymphocyte activation [52].

The incorporation of pH-responsive inorganic components into nanoparticles is a robust approach to promote the release of antigens in APCs because they can generate CO_2_ and/or NH_3_ in acidic conditions which leads to the rupture of antigen-loaded nanoparticles (Figure 4j). In comparison with the degradation of polymers, the disintegration is more readily available for nano-sized inorganic components in mild acidic conditions. For example, ammonium bicarbonate (NH_4_HCO_3_) was encapsulated in PLGA nanoparticles and could react with hydrogen ions in endosomes and lysosomes that produce NH_3_ and CO_2_, mediating the dissociation of nanoparticles and the rapid release of encapsulated OVA to cytoplasm [30]. Similarly, PLGA-sodium bicarbonate (NaHCO_3_) hybrid nanoparticles have been utilized to deliver an agonist 522 to stimulate the maturation of DCs and the secretion of pro-inflammatory cytokines. The incorporation of bicarbonate salts into PLGA nanoparticles enables 33-fold higher loading of the hydrophobic agonist and the rapid rapture of nanoparticles at acidic pH, resulting in an increased expression of co-stimulatory molecules and improved antigen presentation [53].

Metal-organic frameworks (MOFs) have acid-labile metal–ligand bonds and are therefore able to respond to the acidic environment endo/lysosome (Figure 4k). OVA was incorporated into the frameworks during the synthesis of MOF using Eu and guanine monophosphate (GMP). The OVA-loaded MOFs were further coated with CpG as an endosomal-acting oligonucleotide adjuvant. The MOF-based nanocarriers show high loading of antigens. Moreover, the coordination of Eu and GMP is interrupted under pH 5.0 which results in the rapid degradation of MOF and facilitates the endosomal escape and cytosol release of antigens [54].

## 4. Conclusions and Perspectives

pH-responsive nanoparticles show tremendous potential in cancer immunotherapy because they are capable of targeting the acidic microenvironment of tumor tissues and organelles in cells, therefore, reducing the off-targeting toxic side effects and improving therapeutic efficacy. Current pH-responsive nanoparticles for cancer immunotherapy are constructed via distinct mechanisms involving the protonation/ionization of pH-sensitive groups or the break of acid-labile covalent bonds. The key components of pH-responsive nanoparticles and their delivery payloads as well as the related disease models in this review are summarized in Table 1. The protonation/ionization varies with pH, leading to the change of surface charges or the hydrophilicity/hydrophobicity balance of nanoparticles, consequently promoting the accumulation of nanoparticles and release of payloads in target sides. The acid-cleavable bonds have been incorporated into the backbone of polymers or the linker of PEG and the backbone in pH-responsive nanoparticles. The pH-sensitive linkers are stable at physiological pH, affording nanoparticles’ great serum stability upon protection by PEG corona while their cleavage at acidic pH leads to the detachment of the PEG corona and the subsequent exposure of positively charged or targeting groups on nanoparticles, resulting in the enhanced accumulation of nanoparticles in tumors or target cells. The degradation of polymer backbone at acidic pH results in the dissociation of nanoparticles, promoting the release of immunotherapeutic drugs into target sides. Incorporating pH-responsive inorganic components into nanoparticles is a robust approach to the construction of pH-sensitive nanoparticles for cancer immunotherapy because they are able to rapidly respond to the acidic environment and trigger the instant disintegration of nanoparticles at low pH.

The convergence of pH-responsive nanotechnology and immunotherapy provides a promising strategy for improving the unsatisfactory efficacy and reducing the off-target side effects in cancer treatments. Despite the substantial advances in animal models, challenges remain in the clinical translation of cancer immunotherapy with pH-responsive nanoparticles. The therapeutic efficacy should be further evaluated in clinical relevant tumor models. Moreover, clinically translatable materials should be developed for the construction of pH-responsive nanoparticles to meet the strict requirements in clinical use. With the rapid progresses in material chemistry, immunology, and nanotechnology, pH-responsive nanoparticles are expected to exert a significant role in cancer immunotherapy in the near future.

## Figures and Tables

**Table 1:** Selected pH-responsive nanoparticle systems for cancer immunotherapy.

	pH-Responsive Components	Immunotherapeutic Drugs	Disease Models	Refs.
pH-responsive protonation/ionization	Amines	3pRNA	CT26 colon cancer	[33]
		Photosensitizer, siPD-L1	B16-F10 melanoma	[34]
		Luciferase mRNA	C57BL/6 mice	[17]
		OVA	C57BL/6 mice, INF-α/βR^−/−^mice et al.	[38]
		BLZ-945, Pt-prodrug	B16 melanoma, CT26 colon cancer	[43]
		IL-12	B16-F10 tumor	[41]
		CpG ODN, anti-IL-10 ODN, anti-IL-10 receptor ODN	Hepa 1–6 tumor	[42]
	Carboxyl groups	OVA	E.G7-OVA tumor	[39]
	Amines and Carboxyl groups	PTX and IL-2	Murine melanoma	[40]
Acid-liable bonds	Schiff base bonds	OVA	E.G7 tumor	[44]
	Hydrazone bonds	OVA	Melanoma	[29]
		TLR7/8 agonist, R848, DOX	4T1 tumor	[49]
		/	C57BL/6 mice	[46]
	Amide bonds	siPD-L1, photosensitizer	B16-F10 tumor	[27]
		Oxaliplatin, NLG919	4T1 tumor	[47]
	Ester bonds	EGFP and luciferase mRNA	Multiple cell lines, BALB/c mice	[45]
		gp100, OVA et al., and JSI-124	B16-F10 tumor	[50], [51]
	Benzamide bonds	siVEGF and siPIGF	4T1, lung metastasis tumor	[48]
	NH_4_HCO_3_, NaHCO_3_	OVA, agonist 522	C57BL/6 mice, B16-F10 tumor	[30,53]
	Metal–ligand bonds	OVA, CpG	B16 melanoma	[54]
